# Botulinum neurotoxin type-A enters a non-recycling pool of synaptic vesicles

**DOI:** 10.1038/srep19654

**Published:** 2016-01-25

**Authors:** Callista B. Harper, Andreas Papadopulos, Sally Martin, Daniel R. Matthews, Garry P. Morgan, Tam H. Nguyen, Tong Wang, Deepak Nair, Daniel Choquet, Frederic A. Meunier

**Affiliations:** 1 The University of Queensland, Queensland Brain Institute, Clem Jones Centre for Ageing Dementia Research, Brisbane, Queensland 4072, Australia; 2 The University of Queensland, Queensland Brain Institute, Brisbane, Queensland 4072, Australia; 3 The University of Queensland, Centre for Microscopy and Microanalysis, Brisbane, Queensland 4072, Australia; 4 Interdisciplinary Institute for Neuroscience, The University of Bordeaux, Bordeaux, 33000, France; 5 Centre for Neuroscience, Indian Institute of Science, Bangalore, 560012, India

## Abstract

Neuronal communication relies on synaptic vesicles undergoing regulated exocytosis and recycling for multiple rounds of fusion. Whether all synaptic vesicles have identical protein content has been challenged, suggesting that their recycling ability may differ greatly. Botulinum neurotoxin type-A (BoNT/A) is a highly potent neurotoxin that is internalized in synaptic vesicles at motor nerve terminals and induces flaccid paralysis. Recently, BoNT/A was also shown to undergo retrograde transport, suggesting it might enter a specific pool of synaptic vesicles with a retrograde trafficking fate. Using high-resolution microscopy techniques including electron microscopy and single molecule imaging, we found that the BoNT/A binding domain is internalized within a subset of vesicles that only partially co-localize with cholera toxin B-subunit and have markedly reduced VAMP2 immunoreactivity. Synaptic vesicles loaded with pHrodo-BoNT/A-Hc exhibited a significantly reduced ability to fuse with the plasma membrane in mouse hippocampal nerve terminals when compared with pHrodo-dextran-containing synaptic vesicles and pHrodo-labeled anti-GFP nanobodies bound to VAMP2-pHluorin or vGlut-pHluorin. Similar results were also obtained at the amphibian neuromuscular junction. These results reveal that BoNT/A is internalized in a subpopulation of synaptic vesicles that are not destined to recycle, highlighting the existence of significant molecular and functional heterogeneity between synaptic vesicles.

Botulinum neurotoxin type-A (BoNT/A) is a highly potent neurotoxin that acts at the level of presynaptic motor nerve terminals, binding both gangliosides and a protein acceptor in order to be endocytosed^1^,^2^,^3^,^4^. Following translocation from the acidic endosomal compartment, the BoNT/A light chain, which displays endopeptidase activity, cleaves SNAP-25, thereby inducing flaccid paralysis^5^,^6^,^7^,^8^,^9^. The protein receptor of BoNT/A is the synaptic vesicle protein, SV2^3^,^10^, and we and others have found that BoNT/A predominantly enters synaptic vesicles^1^,^10^,^11^. Recently, BoNT/A was also shown to undergo retrograde transport in autophagosomes^12^ although how these retrograde carriers are generated at the presynapse has not yet been determined. Given that BoNT/A is mainly internalized in synaptic vesicles, it is possible that at least some of the BoNT/A-containing vesicles might not recycle but rather comprise a specific pool of synaptic vesicles with a retrograde trafficking fate.

The molecular content of synaptic vesicles and the role of these proteins in vesicular trafficking have been comprehensively defined^13^,^14^. On average, 70 copies of VAMP2, a critical vesicular protein involved in exocytic fusion, can be found on synaptic vesicles^13^,^14^. However, whether all synaptic vesicles have similar protein content has recently been challenged^15^. This raises the possibility that functional heterogeneity may exist within synaptic vesicle pools.

As a portion of BoNT/A follows a retrograde route, we hypothesized that it could be internalized into a selective pool of synaptic vesicles whose fate is not to recycle but to undergo retrograde trafficking. We therefore asked whether BoNT/A is internalized into vesicles distinct from those that undergo local recycling.

Using quantitative single molecule localization microscopy, we discovered that the majority of vesicles containing the receptor-binding domain of BoNT/A (BoNT/A-Hc) had a reduced VAMP2 content. Moreover, destaining experiments carried out in cultured hippocampal neurons loaded with pHrodo labeled BoNT/A-Hc revealed a reduction in the ability of the BoNT/A-Hc-containing vesicles to undergo multiple rounds of exocytosis. Similar results were obtained when we compared FM1-43 and BoNT/A-Hc destaining at the amphibian neuromuscular junction. Overall, our results demonstrate that BoNT/A-Hc-containing synaptic vesicles are non-recyclable, suggestive of a distinct functional role. They could represent precursors whose fate is to generate retrograde carriers such as autophagosomes.

## Results

**Figure 1:**
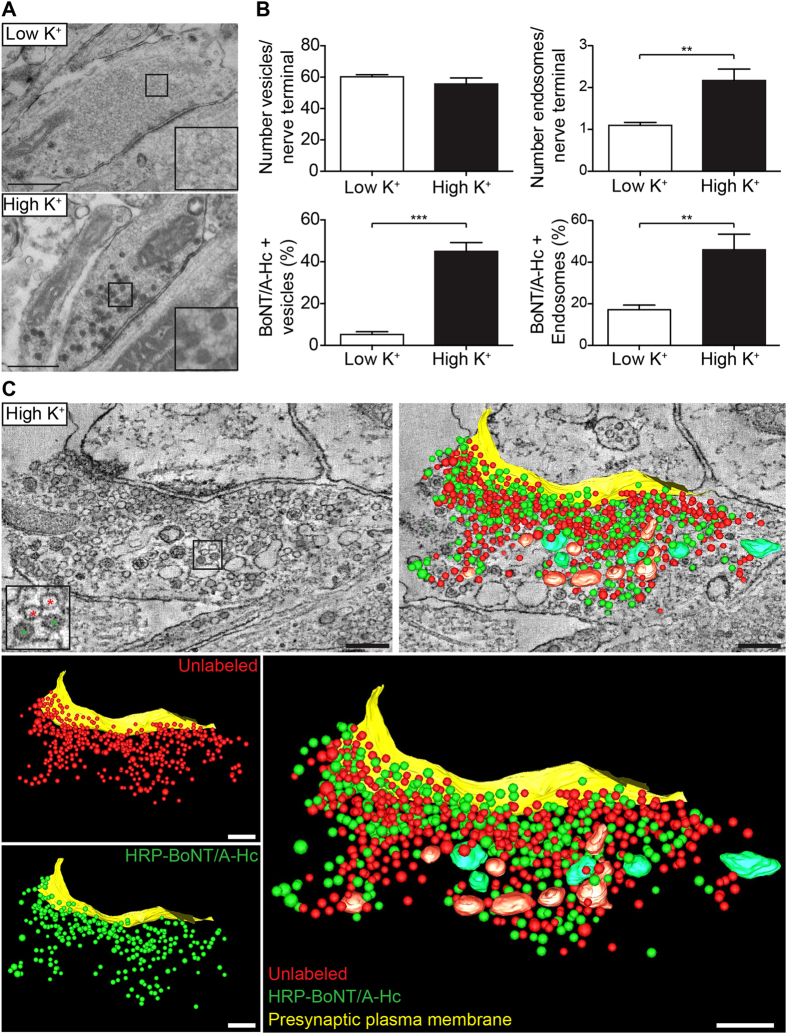
BoNT/A-Hc is internalized in a subpopulation of synaptic vesicles in presynaptic hippocampal nerve terminals. Hippocampal neurons were incubated with HRP-BoNT/A-Hc (10 μg/ml) for 5 min in low K^+^ or high K^+^ buffer, fixed and processed for electron microscopy. (**A**) Representative images of HRP-BoNT/A-Hc endocytosis into control or high K^+^-treated presynaptic regions (50 nm sections). Scale 500 nm. (**B**) The total number of vesicles (40–80 nm diameter) and endosomes (>80 nm) in synaptic regions were quantified, together with the percentage of compartments containing HRP-BoNT/A-Hc. Mean ± sem of 4–6 independent neuron preparations (>40 synapses analyzed/preparation). **p < 0.01, ***p < 0.001. (**C**) An optical slice and three-dimensional reconstruction of a representative presynaptic region that has endocytosed HRP-BoNT/A-Hc (green asterisks in enlargement). HRP-BoNT/A-Hc-containing organelles are distributed randomly in relation to non-labeled compartments and the presynaptic plasma membrane. Scale 200 nm.

We first tested whether BoNT/A was internalized into endocytic synaptic vesicles distinct from those that undergo local recycling by characterizing the trafficking of BoNT/A in cultured hippocampal neurons that were incubated with recombinant BoNT/A-Hc^1^ labeled with horseradish peroxidase (HRP) at 37 °C. As previously demonstrated^1^, HRP-BoNT/A-Hc uptake was significantly increased upon depolarization (Fig. 1A,B), with 45% of synaptic vesicles containing the toxin (Fig. 1A,B). Our stimulation protocol did not affect synaptic vesicle numbers^16^, although a slight increase in the number of endosomes was detected (Fig. 1A,B). Electron tomography further highlighted the distribution of BoNT/A-Hc-containing vesicles and endosomes throughout the nerve terminal (Fig. 1C, Supplementary Movie S1).

**Figure 2:**
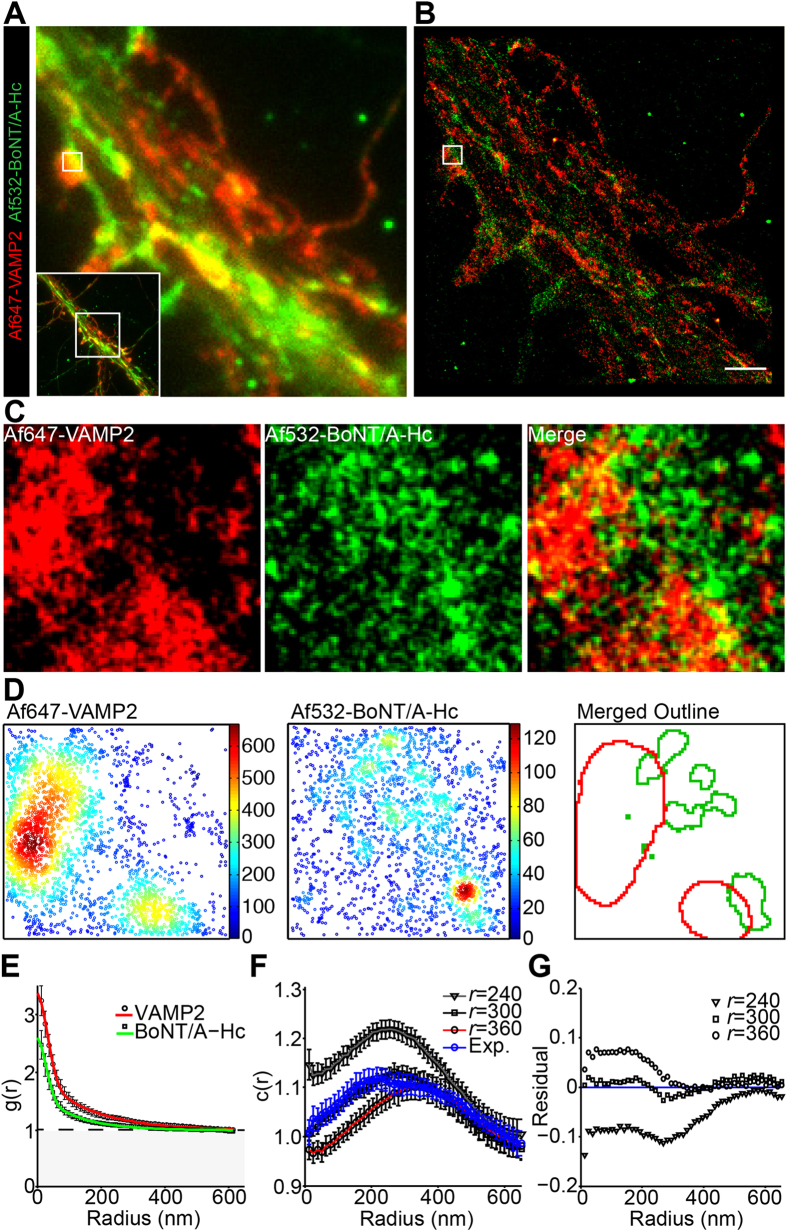
Segregation of Af532-BoNT/A-Hc clusters from VAMP2 in hippocampal nerve terminals. Hippocampal neurons were incubated with Af532-BoNT/A-Hc (150 nM) for 5 min in high K^+^ buffer, fixed and immunolabeled using a VAMP2 antibody, prior to GSDIM imaging and processing. A representative low-resolution image (**A**) and corresponding reconstructed super-resolution localization image (**B**) are shown. Scale 2 μm. (**C**) Enlargement of the synaptic region indicated in **(B)**. (**D**) Cluster maps generated from Ripley’s K-function and outlines of interpolated maps are shown for the enlargement. (**E**) Mean (±sem) auto-correlation functions of each channel fitted using equation (4) (21 regions of interest (ROIs) from 2 independent experiments). (**F**) Cross-correlation function of Af647-VAMP2 and Af532-BoNT/A-Hc at VAMP2-enriched synaptic regions (Exp.) (21 regions of interest from 2 independent experiments). Cross-correlation functions were also used to analyze simulated data of clusters that were separated by varying radii (indicated by *r*). (**G**) Residuals were calculated by subtracting the cross-correlation values of simulated data from the experimental data.

Given that fewer than half of the synaptic vesicles in the nerve terminal contained BoNT/A-Hc, we examined whether this population differed from canonical synaptic vesicles that undergo regulated exocytosis. As VAMP2 is required to promote fusion and is thought to be present on the majority of synaptic vesicles, cells were incubated with Alexa Fluor 532 labeled BoNT/A-Hc (Af532-BoNT/A-Hc) then fixed and immunolabeled using an antibody against VAMP2, followed by an Alexa Fluor 647 labeled secondary antibody. Although epifluorescence imaging revealed colocalization between the two markers (Fig. 2A), nanoscopy by ground state depletion followed by individual molecule return (GSDIM)^17^ revealed that VAMP2 labeling was largely separate from that of BoNT/A-Hc at synapses (Fig. 2B,C). Cluster maps created by calculating Ripley’s K function^18^,^19^ revealed large, dense clusters of VAMP2 molecules adjacent to smaller clusters of BoNT/A-Hc molecules (Fig. 2D).

We used an auto-correlation function^20^,^21^ to quantify the distribution of each protein individually (Fig. 2E, Table 1). Cluster characteristics (Table 1) were determined by fitting the auto-correlation values to a function accounting for over-counting of localizations that have a finite resolution. Both proteins exhibited a large cluster size (Table 1), likely to represent a conglomerate of synaptic vesicles. The number of VAMP2 molecules found per synapse was lower than previously described, which is likely to result from the small proportion of molecules detected during single molecule imaging and the high density of the molecules^14^,^15^,^22^ (Table 1). Cross-correlation was then used to quantify the distribution of detectable VAMP2 relative to BoNT/A-Hc^20^,^21^. A peak in the average cross-correlation values was observed at approximately 200 nm (Fig. 2F), suggesting that the distance between clusters of VAMP2 and BoNT/A-Hc molecules was similar across multiple analyzed regions. Simulations of displaced clusters at set radii confirmed that the centers of the BoNT/A-Hc and VAMP2 clusters were separated by approximately 300 nm on average (Fig. 2F,G). However, some overlap between BoNT/A-Hc and VAMP2 could be observed (Fig. 2C), suggesting that some regions did exhibit partial colocalization. To address this issue, cluster maps generated using Ripley’s K-function were analyzed. Although Pearson’s coefficient indicated little colocalization (0.076 ± 0.027) due to the difference in cluster size and spatial distribution, Mander’s coefficient, which provides the proportion of pixels that overlap with the second channel, confirmed some overlap between the clusters of VAMP2 (0.259 ± 0.043) and BoNT/A-Hc (0.323 ± 0.046) (Fig. 2D).

**Figure 3:**
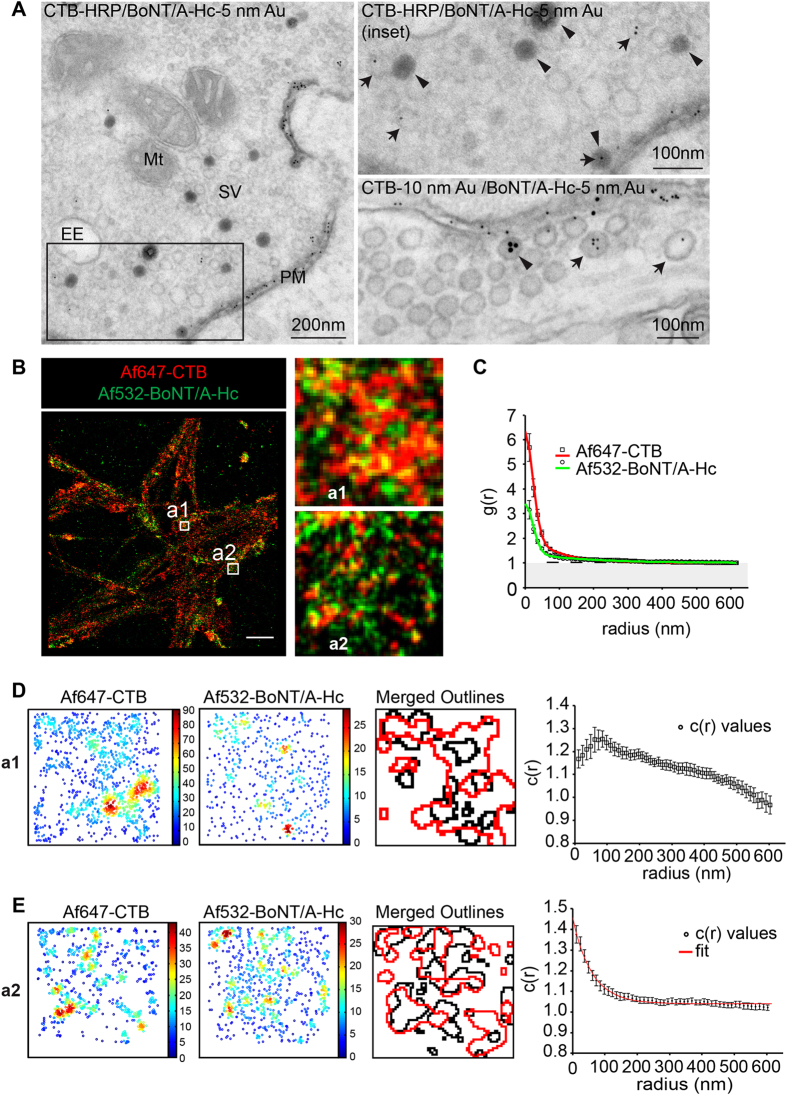
Af532-BoNT/A-Hc is partially segregated from Af647-CTB following internalization in hippocampal nerve terminals. (**A**) Hippocampal neurons were incubated with BoNT/A-Hc labeled with 5 nm gold (BoNT/A-Hc-5 nm Au) and CTB-HRP or CTB-10 nm Au for 5 min in the presence of high K^+^ prior to fixation and processing for electron microscopy. Mt, mitochondria; SV, synaptic vesicle; PM, plasma membrane; EE, early endosome. The arrowheads show CTB-HRP or CTB-10 nm Au, whilst the arrows indicate BoNT/A-Hc-5 nm Au. Af647-CTB (35 ng/ml) and Af532-BoNT/A-Hc (150 nM) were added to cultured neurons in the presence of high K^+^ for 5 min prior to fixation and GSDIM imaging. The data was then processed to provide coordinates and images. A representative reconstructed localization image is shown (**B**), along with enlargements where CTB and BoNT/A-Hc appear predominantly segregated (a1) or colocalized (a2) Scale 2 μm. (**C**) Mean (±sem) auto-correlation functions of each channel fitted using equation (4) (36 ROIs from 3 independent experiments). Cross-correlation functions of Af647-CTB and Af532-BoNT/A-Hc could be split into 2 trends (**D** and **E**). Af647-CTB and Af532-BoNT/A-Hc shows regions where they are mainly segregated (**D**), which is represented by the enlargement (a1). Cluster maps generated from Ripley’s K-function and outlines of interpolated maps are shown, along with mean (±sem) cross-correlation functions calculated for these segregated regions, which show peak correlation at a radius of approximately 86 nm (18 ROIs from 3 independent experiments). (**E**) The remaining ROIs analyzed predominantly show colocalization of Af647-CTB and Af532-BoNT/A-Hc as shown in enlargement (a2). Cluster maps of the enlargement (a2) were produced and the mean (±sem) cross-correlation functions of the colocalized regions are fit well by equation (10) (18 ROIs, 3 independent experiments).

To confirm that the observed segregation between BoNT/A-Hc and VAMP2 was not a result of the imaging technique used, the distribution of internalized BoNT/A-Hc relative to a classical endocytic marker, cholera toxin B-subunit (CTB), was characterized. To first assess whether the two proteins were internalized in the same vesicles, electron microscopy was carried out using BoNT/A-Hc conjugated to 5 nm gold (BoNT/A-Hc-5 nm Au) and CTB conjugated to either 10 nm gold (CTB-10 nm Au) or HRP (CTB-HRP) (Fig. 3A). As with BoNT/A-Hc, the labeled CTB could be observed in small synaptic vesicles, however little colocalization with BoNT/A-Hc-5 nm Au was observed. Quantification of the distribution of CTB-HRP and BoNT/A-Hc-5 nm Au showed that while only 4.4 ± 1.6% of vesicles labeled with CTB-HRP contained BoNT/A-Hc-5 nm Au, a significant portion of BoNT/A-Hc-5 nm Au vesicles also labeled with CTB-HRP (30.0 ± 6.1%) (mean ± sem, n = 3 independent experiments, >20 individual synapses/experiment). While fewer BoNT/A-Hc-5 nm Au-containing vesicles were observed relative to HRP-BoNT/A-Hc-containing vesicles, our result confirmed that BoNT/A-Hc is only present in a subset of endocytosed vesicles.

The distribution of CTB and BoNT/A-Hc observed by electron microscopy was then compared to the distribution of the two proteins obtained by GSDIM. Cultured neurons incubated with Af532-BoNT/A-Hc and Alexa Fluor 647 labeled CTB (Af647-CTB) were fixed and imaged by GSDIM (Fig. 3B). Quantification of the clusters of the individual proteins was carried out using an auto-correlation function and the values were fitted to equation (4) (Table 1). As observed previously, BoNT/A-Hc showed similar cluster dimensions (Table 1), while CTB was present in smaller clusters with a radius of 85 nm (Table 1).

Examination of the nerve terminals positive for BoNT/A-Hc labeling, showed partial overlap between the two markers (Fig. 3B). The level of co-clustering between CTB and BoNT/A-Hc was quantified using a cross-correlation function. The cross-correlation values for each of the regions analyzed showed two trends and the data was split according to their distribution (Fig 3D,E). The cross-correlation values for half of the regions indicated that the clusters of CTB and BoNT/A-Hc were predominantly segregated by approximately 85 nm (Fig. 3D), while the remaining ROIs exhibited cross-correlation values indicative of co-clustering of CTB and BoNT/A-Hc (Fig. 3E). Fitting of the latter cross-correlation values to a single exponential equation (7) gave a cluster size of 58.9 ± 5.9 nm. This data supports the low level of colocalization observed by electron microscopy and shows that while there is some level of co-clustering between BoNT/A-Hc and CTB, there is significant separation of the two endocytic markers. Overall, this data indicates that BoNT/A-Hc is taken up in a specific subpopulation of endocytic vesicles, only a fraction of which contain CTB.

**Figure 4:**
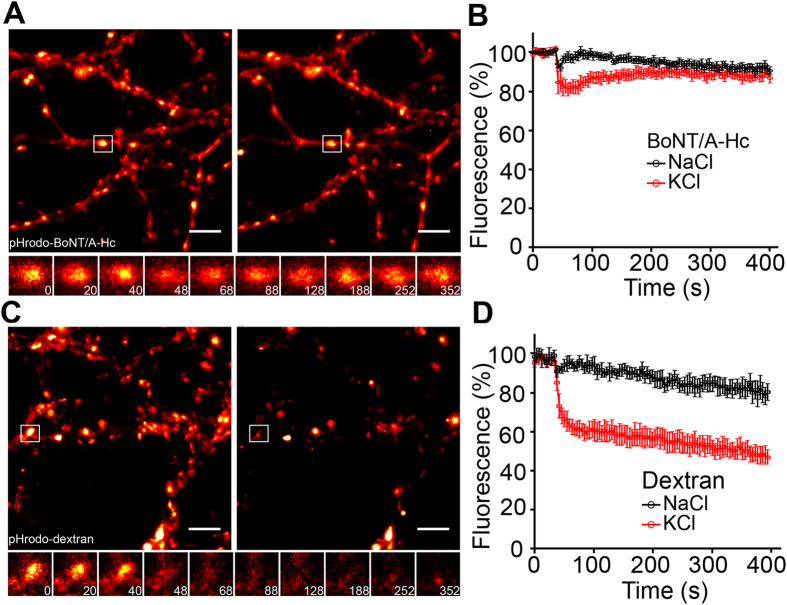
BoNT/A-Hc-containing vesicles exhibit reduced ability to undergo exocytosis at the presynaptic nerve terminal of hippocampal neurons. Hippocampal neurons (14–17 days *in vitro*) were loaded with either pHrodo-BoNT/A-Hc (300 nM) (**A,B**) or pHrodo-dextran (0.1 mg/ml) (**C,D**) during a 2 min high K^+^ stimulation. Neurons were left to recover for 12–15 min prior to a second stimulus (30 mM KCl or NaCl as control) during which a time-lapse movie was acquired. (**A**,**C**) Representative nerve terminals prior to the second KCl stimulus and (**B**,**D**) 400 s following the stimulus. Enlargements show the response of a representative nerve terminal to the second stimulation over time. Scale 5 μm. (**E,F**) Normalized fluorescence of nerve terminals loaded with pHrodo-BoNT/A-Hc (*n* = 8 experiments from 2 independent preparations) (**E**) or pHrodo-dextran (*n* = 4 experiments from 2 independent preparations) (**F**) in response to a KCl stimulation or NaCl control. Data are plotted as mean ± sem.

Given the essential role of VAMP2 in exocytosis^14^, our data showing reduced VAMP2 levels in BoNT/A-Hc-containing vesicles suggest that they may exhibit a reduced ability to undergo recycling. We therefore tested the ability of BoNT/A-Hc-containing vesicles to undergo regulated exocytosis using a pH-sensitive dye, pHrodo, which when conjugated to BoNT/A-Hc has a pKa of 7.03 making it suitable for studying synaptic vesicle recycling (Supplementary Fig. S1). The dye displays minimal fluorescence at physiological pH that increases substantially upon acidification to pH 5.5 found in a synaptic vesicle lumen^23^ (Supplementary Fig. S1). Upon stimulation of hippocampal neurons pre-loaded with pHrodo-BoNT/A-Hc, we could only detect a slight, transient decrease in fluorescence (~10% below control), which subsequently returned to baseline levels, indicating that very few pHrodo-BoNT/A-Hc-positive synaptic vesicles undergo exocytic fusion (Fig. 4A,B). Fitting of the recovery to a single exponential gave an endocytic time constant of 32 s. In sharp contrast, terminals loaded with pHrodo-dextran (10,000 MW) (Fig. 4C,D) showed a dramatic decrease (~50%) in fluorescence upon stimulation, demonstrating significant fusion of pre-loaded synaptic vesicles with the plasma membrane and release of dextran into the extracellular space^23^,^24^.

We confirmed that endocytic compartments labeled by pHrodo-BoNT/A-Hc and pHrodo-dextran could be detected by eliciting a rapid change in pH using NH_4_Cl (Supplementary Fig. S2a,b). We also investigated whether the response of pHrodo dye to alterations in pH could influence the kinetics of our measurements. We found that the pH-dependent change in fluorescence was rapid and occurred within milliseconds, suggesting that this was not the case (Supplementary Fig. S2c).

**Figure 5:**
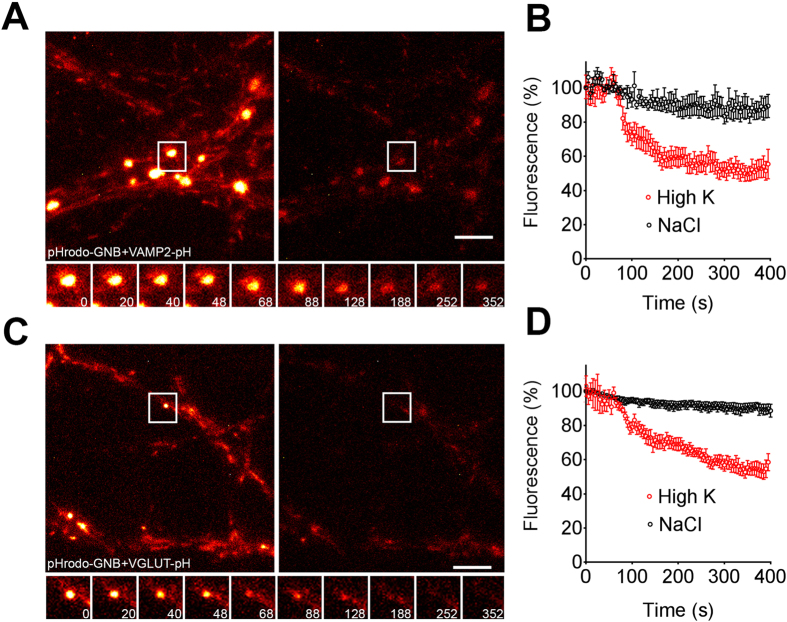
VAMP2-pHluorin and vGlut-pHluorin-containing vesicles labeled with pHrodo-GNBs undergo repeated rounds of exocytosis at the presynaptic nerve terminal of hippocampal neurons. Hippocampal neurons (14–17 days *in vitro*) transfected with either VAMP2-pHluorin (**A**,**B**) or vGlut-pHluorin (**C**,**D**) were loaded with pHrodo-GNBs during a 2 min high K^+^ stimulation. Neurons were left to recover for 12–15 min prior to a second stimulus during which a time-lapse movie was acquired (30 mM KCl or NaCl as control). (**A**,**C**) Representative nerve terminals prior to the second KCl stimulus and 400 s following the stimulus. Enlargements show the response of a representative nerve terminal to the second stimulation over time. Scale 5 μm. (**B**,**D**) Normalized fluorescence of nerve terminals loaded with pHrodo-GNBs in cells transfected with VAMP2-pHluorin (*n* = 5) (**B**) or vGlut-pHluorin (*n* = 4) (**D**) in response to KCl or NaCl. Data are plotted as mean ± sem.

To establish the level of pHrodo fluorescence lost during vesicle recycling upon stimulation, we performed an experiment using hippocampal neurons expressing either vGlut-pHluorin or VAMP2-pHluorin. These neurons were briefly stimulated in the presence of pHrodo-tagged nanobodies against GFP in order to promote their activity-dependent uptake into recycling synaptic vesicles, then washed and imaged during another round of stimulation (Fig. 5A–D). Our results show that contrary to pHrodo-BoNT/A-Hc, the fluorescence decrease detected with pHrodo-anti-GFP nanobodies associated with either vGLUT-pHluorin or VAMP2-pHluorin was more pronounced, as expected for the recycling nature of these pools of synaptic vesicles (Fig. 5A,D). Our data strongly suggests that pHrodo-BoNT/A-Hc is predominantly internalized in a non-recycling compartment.

**Figure 6:**
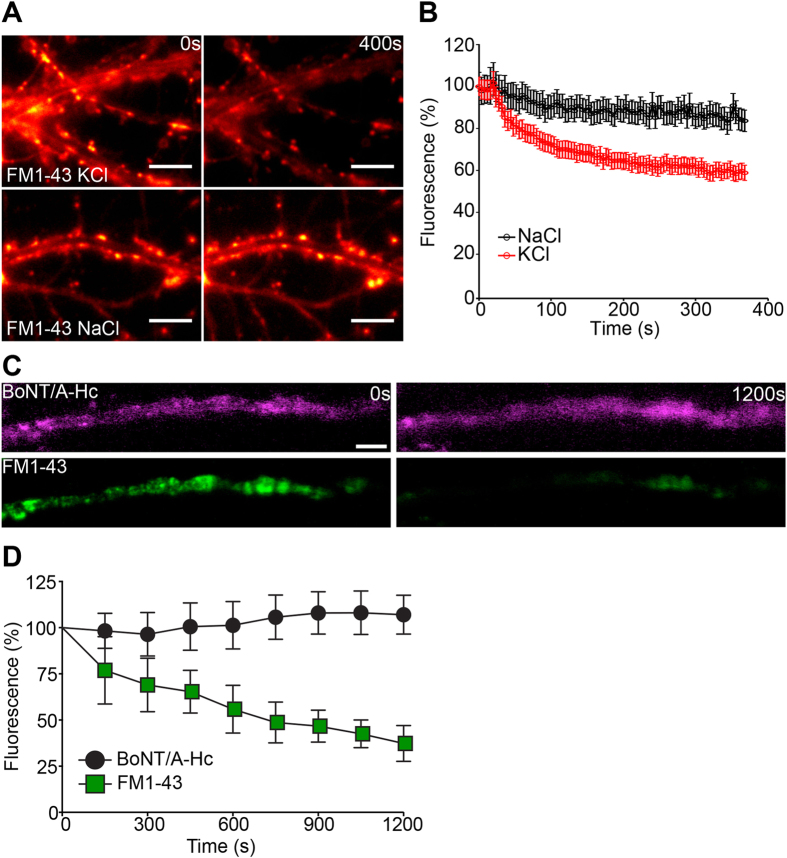
BoNT/A-Hc is retained in non-releasable vesicles upon a secondary stimulus. (**A**,**B**) Cultured hippocampal neurons were incubated with FM1-43 (4 μM) for 2 min in the presence of high K^+^ and following a 12–15 min recovery were subjected to a secondary stimulus of KCl (30 mM) or NaCl (30 mM). Representative before and after images are shown (**A**). (**B**) Quantification of a representative experiment showing the change in fluorescence during stimulation. Data are plotted as mean ± sem (*n* = 32–35 nerve terminals from a representative experiment). Amphibian neuromuscular junction preparations were incubated with Atto647N-BoNT/A-Hc (800 nM) for 20 min in the presence of high K^+^ Ringer’s solution. FM1-43 (5 μM) was applied in the last 3 min. Preparations were washed with low K^+^ Ringer’s solution and left to recover for 20 min. Destaining was induced by replacing the solution with high K^+^ Ringer’s solution and confocal time-lapse imaging of nerve terminals was carried out. (**C**) Representative time-lapse images of a nerve terminal showing destaining of FM1-43 but not Atto647N-BoNT-A-Hc in response to the secondary high K^+^ stimulus. Scale 10 μm. (**D**) Rate of destaining of Atto647N-BoNT-A-Hc (magenta) and FM1-43 (green) in response to the secondary high K^+^ stimulus (*n* = 3 independent preparations).

We carried out similar experiments at amphibian motor nerve terminals, comparing the change in fluorescence of Atto647N-BoNT/A-Hc and that of the lipophilic dye, FM1-43. We first confirmed that FM1-43 could be destained in hippocampal neurons using our stimulation protocol (Fig. 6A,B). Stimulation of amphibian motor nerve terminals also resulted in a destaining of FM1-43, whereas no alteration to the fluorescence or distribution of Atto647N-BoNT/A-Hc could be observed in the same nerve terminals (Fig. 6C,D). This data indicates that BoNT/A-Hc is also retained in non-recycling vesicles at the amphibian motor nerve terminal.

## Discussion

Together, our results indicate that a population of BoNT/A-Hc-positive endocytic vesicles are unable, or have a much-reduced ability to recycle in hippocampal and amphibian motor nerve terminals. Our GSDIM data suggest that their inability to undergo recycling could stem from reduced VAMP2 levels. Such heterogeneity in the number of VAMP2 molecules on synaptic vesicles^15^ could result from VAMP2 being recycled through a synaptophysin-dependent route^25^, amongst other possibilities. Our functional data demonstrate that the endocytic pool of synaptic vesicles loaded with pHrodo-dextran readily undergo exocytosis. This result is consistent with a previous study also using pHrodo-dextran to label synaptic vesicles in isolated rod synaptic ribbons^23^. Such recycling is in sharp contrast to that observed for pHrodo-BoNT/A-Hc, which showed a strikingly small fluorescent response to stimulation, indicating that this pool of endocytic vesicles did not have the ability to undergo further rounds of exocytosis.

These data indicate that the majority of pHrodo-dextran enters the recycling pool of synaptic vesicles. In hippocampal neurons, this population comprises less than 20% of the total population of synaptic vesicles when characterized using HRP^26^,^27^. BoNT/A-Hc therefore enters a much larger pool of synaptic vesicles (45%, see Fig. 1) compared to the recycling pool of synaptic vesicles^26^,^27^, although this discrepancy is at least partially due to the inefficient labeling observed with fluid-phase markers. Our result is in good agreement with a similarly large distribution of BoNT/A-Hc-labeled synaptic vesicles detected in mouse motor nerve terminals following subcutaneous injection^6^. In addition, our results reveal that CTB is internalized in an even larger population of synaptic vesicles, a fraction of which are also positive for BoNT/A-Hc. More work is needed to establish whether it is the CTB-positive or negative population of BoNT/A-Hc vesicles that has a reduced ability to recycle. How and why BoNT/A-Hc is limited to a non-recyclable pool also remains to be established. The size of the pore could influence the toxin’s access to such a pool. Alternatively, BoNT/A-Hc could manipulate the recycling ability of endocytic synaptic vesicles. Future experiments will be needed to precisely quantify the level of overlap between the pool of vesicles labeled with BoNT/A-Hc and recycling synaptic vesicles. Other VAMP isoforms, namely VAMP1, could be involved in BoNT/A-Hc trafficking as VAMP1 is present both at the neuromuscular junction^28^ and a small subpopulation of hippocampal neurons^29^. More work will be required to investigate whether BoNT/A-Hc enters/is sorted to VAMP1-positive synaptic vesicles.

We have recently demonstrated that a large proportion of internalized BoNT/A-Hc undergoes axonal retrograde trafficking in autophagosomes in living hippocampal neurons cultured in microfluidic devices and in motoneurons *in vivo*^12^. These retrograde carriers may be generated from the endocytic vesicles containing BoNT/A-Hc. However, some BoNT/A-Hc remains trapped at the nerve terminal^30^, suggesting that not all BoNT/A-Hc-containing vesicles undergo long-range retrograde transport. Importantly, we found that the majority of BoNT/A-Hc-positive cargoes are targeted for lysosomal degradation upon reaching the cell body and only a minor component of the toxin manages to escape this dead-end trafficking fate and intoxicate neighbouring neurons^12^. Future experiments will be needed to examine the nature of all the retrograde carriers generated and identify those responsible for such retrograde intoxication.

We observed that the small signal in pHrodo-BoNT/A-Hc fluorescence elicited by stimulation rapidly returned to baseline, indicating that the low level of pHrodo-BoNT/A-Hc, which does undergo recycling is rapidly internalized and re-acidified. This suggests that upon stimulation, BoNT/A-Hc, which is re-exposed during exocytosis, is rapidly retrieved, raising the possibility that the toxin fragment is still bound to its receptor. While our observed time constant of 32 s is slightly slower than that previously observed (19–23 s)^31^,^32^, large variations in retrieval rates between cells have been reported^33^, which could account for this apparent discrepancy.

BoNT/A binds the synaptic vesicle protein, SV2^10^. Given the ability of SV2 to undergo recycling^31^,^34^, it would be of interest to investigate whether BoNT/A-Hc remains bound to its receptor in these non-recyclable vesicles and whether SV2-positive vesicles have a reduced ability to recycle. More generally, key steps in the endocytic pathway leading BoNT/A-Hc to enter the retrograde axonal pathway will need to be identified^12^ and whether temperature can affect the sorting of BoNT/A-Hc by altering the balance between the retrograde and recycling pathway. Although previous studies have identified differences in the ability of synaptic vesicles to undergo exocytosis^35^,^36^, our study provides the first evidence for functional heterogeneity in the ability of synaptic vesicles to recycle. While the role of these non-exocytic, VAMP2-negative vesicles is still unclear it is tempting to speculate that they may be involved in generating retrograde carriers. Given the ability of BoNT/A to undergo retrograde transport^12^,^30^, these small BoNT/A-Hc-positive vesicles that contain 80% of internalized BoNT/A-Hc^1^ could represent an early endocytic step leading to the formation of retrograde carriers including autophagosomes^12^,^30^. However, further work will be needed to investigate this possibility.

## Materials and Methods

### Antibodies, fluorescent labels and reagents

The rabbit anti-synaptobrevin-2 (VAMP2) antibody was obtained from Synaptic Systems (#104 202). Alexa Fluor secondary antibodies, together with maleimide dyes, pHrodo-dextran, Alexa Fluor labeled CTB and FM1-43 were purchased from Invitrogen. Atto647N maleimide was obtained from Atto Tec. The remaining reagents were obtained from Electron Microscopy Sciences or Sigma Aldrich unless otherwise specified. The expression, purification and fluorescent labeling of BoNT/A-Hc was carried out as previously described^1^,^37^. BoNT/A-Hc was conjugated to HRP using the EZ-Link Maleimide Activated Horseradish Peroxidase Kit (Thermo Scientific), while BoNT/A-Hc and CTB were conjugated to colloidal gold as described previously^1^. The anti-GFP nanobody (kind gift from Kirill Alexandrov) was conjugated to pHrodo-NHS-ester.

### Neuronal cultures

Hippocampal neurons were cultured from embryonic age 18 mice or rats with approval from the University of Queensland Animal Ethics Committee as previously described^1^,^38^,^39^. The methods were carried out in accordance with the approved guidelines. Neurons were plated on coverslips or alternatively on glass bottom dishes (MatTek Corporation) with the astroglia seeded on the surrounding plastic.

### Electron microscopy

Primary hippocampal neurons (13–16 days *in vitro*) were incubated with either HRP-BoNT/A-Hc (10 μg/ml), HRP-CTB (10 μg/ml), BoNT/A-Hc-5 nm Au (1:20) or CTB-10 nm Au (1:10), alone or in combination, in either low K^+^ (15 mM HEPES, 145 mM NaCl, 5.6 mM KCl, 2.2 mM CaCl_2_, 0.5 mM MgCl_2_, 5.6 mM D-glucose, 0.5 mM ascorbic acid, 0.1% w/v bovine serum albumin, pH 7.4) or high K^+^ buffer (adjusted to 95 mM NaCl and 56 mM KCl) for 5 min, prior to washing in PBS and fixation with 2.5% glutaraldehyde for 24 h. Following fixation, cells were processed for 3,39-diaminobenzidine (DAB) cytochemistry using standard protocols. Fixed cells were contrasted with 1% osmium tetroxide and 2% uranyl acetate prior to dehydration and embedding in LX-112 resin using a BioWAVE microwave (Pelco, Ted Pelling)^1^. Samples were dehydrated stepwise into ethanol by sequential microwaving at 250 W for 40 s in 70% ethanol (2x), 90% ethanol (2x), and 100% ethanol (3x) before embedding in LX112 resin. For embedding, samples were microwaved at 250 W for 3 min under vacuum in 50% resin (in ethanol) and then twice in 100% resin. The resin was polymerized in a 60 °C oven for 24 h. Sections (50–300 nm) were cut using an ultramicrotome (UC64; Leica). To quantify HRP-BoNT/A-Hc endocytosis, and the colocalization between HRP-CTB and BoNT/A-Hc-5 nm Au, ultrathin (55 nm) presynaptic regions were visualized at 60,000x using a transmission electron microscope (model 1011; JEOL) equipped with a Morada cooled CCD camera and the iTEM AnalySIS software. Membrane-bound compartments within the presynaptic region were counted and scored for the presence or absence of the DAB reaction product or gold particles. The maximum diameter was measured using Image J software. Compartments <80 nm in diameter were classified as synaptic vesicles and compartments >80 nm as endosomes.

For high-resolution tomography, both sides of a thick section (300 nm) were labeled for 8 min with 10 nm colloidal gold diluted 1:10 in H_2_O, followed by three 5 min water washes, to generate fiducial markers. A Tecnai F30 microscope (FEI) was used to image dual-axis tilt series at 1.5° increments from −66° to +66° at an accelerating voltage of 300 kV. Images were captured using an LC-1100 4K x 4K camera (Direct Electron), at a binning of 2, using SerialEM image acquisition software^40^. Tilt series alignments and weighted back projection reconstructions were performed using IMOD software (Boulder Laboratories for 3D Electron Microscopy of the Cell, University of Colorado). Areas to be imaged were selected from five different presynaptic regions in groups of neurites from two independent experiments. Synapses were identified by dense clusters of 40–60 nm vesicles together with the post-synaptic density. Organelles were manually segmented at high fidelity and meshed in 3dmod as previously described^41^. As synaptic vesicles were present as predominantly spherical structures, a scatter point object was used to mark the center of each 3D vesicle and the circumference expanded to the limiting membrane to generate a sphere representing the surface of the vesicle. Compartments containing the HRP-BoNT/A-Hc DAB reaction product were identified by increased density of the intraluminal space. The slicer tool was used in 3dmod, and optical slices were averaged to improve contrast and aid in resolving fine structures. All images were processed using Adobe Photoshop CS3 and figures compiled with Adobe Illustrator CS3.

### Ground state depletion followed by individual molecule return (GSDIM)

Incubation of cultured hippocampal neurons with BoNT/A-Hc was carried out as previously described^1^. Briefly, neurons were washed once with a low K^+^ buffer then incubated with Af532-BoNT/A-Hc (150 nM) and/or Af647-CTB (35 ng/ml) in a high K^+^ buffer for 5 min^1^,^42^. Cells were fixed immediately in 4% paraformaldehyde diluted in Sorensen’s phosphate buffer for 45 min and processed for immunocytochemistry. Immunocytochemistry and single molecule imaging were carried out as described previously^43^. Alexa Fluor 647 was chosen as the label for the secondary antibody as it is one of the most suitable fluorophores for GSDIM^44^. Tetraspeck beads (0.1 μm, Invitrogen) were added to the coverslip to be used as fiducial markers to enable correction of spatial misalignment between channels. Imaging was carried out in a reducing buffer with oxygen scavengers^43^, which was added to the well of a cavity microscope slide prior to sealing the coverslip with silicon. Time-lapse movies were acquired on a commercial GSDIM system (Leica)^17^. Two-channel imaging was carried out sequentially.

### Destaining of hippocampal neurons

Cultured hippocampal neurons (untransfected or transfected with either VAMP2-pHluorin or vGlut-pHluorin) were washed once with a low K^+^ buffer containing 150 mM NaCl, 4 mM KCl, 2 mM MgCl_2_, 2 mM CaCl_2_, 10 mM D-glucose and 10 mM HEPES (pH7.4) before being incubated with pHrodo-dextran (500 μg/ml), pHrodo-BoNT/A-Hc (300 nM), pHrodo-anti-GFP nanobodies (200 nM) or FM1-43 (4 μM) in a high K^+^ buffer (substituted with 109 mM NaCl, 45 mM KCl) containing 10 μM CNQX or NBQX for 2 min. The cultured neurons were then washed extensively with low K^+^ buffer containing 10 μM CNQX/NBQX and allowed to recover for 12–15 min. Cells were subsequently imaged on an inverted microscope (Nikon Eclipse T*i*, 100x, NA 1.49) equipped with a Perfect Focus System and an EMCCD camera (EVOLVE 512 Delta, Photometrics), and controlled using Metamorph software (Molecular Devices). Images were acquired every 4 s, during which the neurons were stimulated by adding 1 M KCl or NaCl in low K^+^ buffer to a final concentration of 30 mM. Alternatively, 50 mM NH_4_Cl was added during imaging to quench the fluorescence of the pHrodo dye.

Time-lapse movies were analyzed using ImageJ. Nerve terminals were selected based on their fluorescence intensity and punctate structure, and their average fluorescent intensity was determined using the plugin, Time Series Analyzer. Background fluorescence from the coverslip was subtracted from the measurements. The change in fluorescence was normalized to the first value for each experiment. The recovery phase of the pHrodo-BoNT/A-Hc was fitted to a single exponential equation to determine the endocytic time constant.

### Fluorescent measurements of pHrodo *in vitro*

The fluorescence intensity of pHrodo-BoNT/A-Hc in response to differing pH *in vitro* was measured by adding the probe to buffers of either pH 5.0, 5.5, 6.5, 7.5, 8.5 or 9.5. In the acidic buffers (pH 5,5.5,6.5), HEPES was replaced with MES, whereas for the basic buffers (pH 8.5, 9.5) it was replaced with TRIS.

To measure the response of pHrodo to rapidly changing pH, pHrodo-BoNT/A-Hc was added to buffers of either pH 4 or 8 and placed on an inverted microscope (Nikon Eclipse T*i*, 100x, NA 1.49). A timelapse series was recorded with an exposure time of 100 ms during which the pH was changed from pH 8 to 4 or from pH 4 to 8 by infusing either HCl or NaOH. The change in fluorescent intensity within a region in the middle of the field of view was then calculated using ImageJ.

### Destaining of the amphibian neuromuscular junction (NMJ)

Destaining experiments were carried out on NMJ preparations of adult toads (Bufo marinus) with approval from the University of Queensland Animal Ethics Committee. The methods were carried out in accordance with the approved guidelines. Toads were euthanized by double pithing. The iliofibularis muscles were dissected and pinned out on a Sylgard-lined organ bath (3 mL capacity) containing low K^+^ amphibian Ringer's solution (116 mM NaCl, 2 mM KCl, 1.8 mM CaCl_2_, 1 mM MgCl_2_, 5 mM HEPES-OH, pH 7.3). NMJ preparations were incubated with Atto647N-BoNT/A-Hc (800 nM) for 20 min in the presence of high K^+^ Ringer’s solution (adjusted to 62 mM NaCl and 56 mM KCl). FM1-43 (5 μM) was applied in the last 3 min of high K^+^ stimulation. Preparations were washed with low K^+^ Ringer’s solution and left to recover for 20 min. Exocytosis was induced by replacing the solution with high K^+^ Ringer’s and dual-color time-lapse imaging of nerve terminals was carried out on a LSM 510 meta confocal microscope (Zeiss).

### GSDIM data analysis

Raw time-lapse movies acquired on a GSDIM microscope were processed using the open source software, Localizer, run from Igor Pro (Wavemetrics)^45^ to obtain coordinates for the individual molecules. To partially account for multiple appearances of molecules, the localizations that appeared within 2 frames and 50 nm of one another were consolidated. Coordinates with a calculated localization precision^46^ between 10 and 40 nm were retained for further analysis. A custom written program in Matlab (The Mathworks) was used to correct for spatial misalignment of the two channels^47^. Datasets were reconstructed with a pixel size of 10 nm and regions of interest were selected from reconstructed 2D histograms to quantify the clustering of the proteins using two methods: Ripley’s K-function was used to generate cluster maps^18^,^19^,^48^, whereas pair-correlation analyses were carried out to quantify the clustering of the proteins^20^,^21^. Both of these were carried out in a custom written Matlab program.

The cluster maps that were generated using Ripley’s K-function were used to visualize the distribution of clusters of VAMP2 and BoNT/A-Hc. The K function is a method used to describe a spatial point pattern at increasing distance scales. The K function is simply:


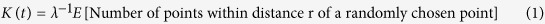


where *λ* is the density of points and *E* is the expectation value. The most commonly used form of the K function is due to Ripley^32^ and can be expressed as:


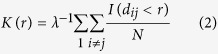


where *I* is the indicator function, *d* is the distance between the points *i* and *j* and *N* is the total number of points. Rather than use the K function directly we used a linearized form that can be expressed as


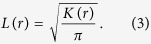


The L function is used to test whether points (in our case single molecule localizations) are consistent with constant spatial randomness, in which case *L(r)* *−* *r* *=* *0*. If *L(r)* *−* *r* is positive this indicates that the distribution of points is more clustered than would be expected for constant spatial randomness. For each analyzed region, the radius at which the L(*r*) value peaked was selected as the threshold for each image. Each localization was assigned a color based on its L value at that radius. The map was then interpolated using a biharmonic spline interpolation method. The images were binarized in ImageJ (NIH)^49^ and overlaid to visualize the distribution of both channels. Alternatively, the colocalization of the binary maps was analyzed using the Just Another Colocalization Plugin (JACoP)^50^.

Pair-correlation analysis is a close analog to convolution and as such can be easily calculated using fast Fourier transforms^20^,^21^. Auto-correlation is the cross-correlation of a signal with itself, and in the context of localization microscopy, auto-correlation values can be fit to the data using equation (4) to obtain the cluster characteristics^20^,^21^:


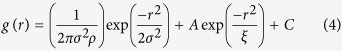


*ρ* is the density of molecules in the image, *ξ* represents the average radius of the clusters or correlation length, while an estimate of the resolution of the image can be given by σ/√2. Α is the amplitude of the exponential decay function and provides an indication of the increase in the density of molecules within the clusters, which is calculated by:


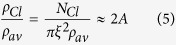


*ρ*_*av*_ is the density of molecules outside the clusters whereas *ρ*_*cl*_ is within. The average number of molecules per cluster can also be approximated by:


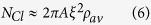


The auto-correlation function has an amplitude of 1/ρ at r = 0; as a result of over-counting localizations that have a finite resolution, this contribution extends to r > 0 by an amount given by the first term in in equation (4). Using cross-correlation avoids the effects of over-counting and allows the study of correlation between channels. Cross-correlation values obtained from clustered data can therefore be fit to an equation that does not account for over-counting:


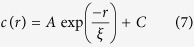


It should be noted that Ripley’s K function is closely related to the pair-correlation analysis, but the former does not account for the effects of over-counting, which becomes propagated across distance scales^20^,^21^,^22^.

### Simulations of localizations

Matlab was used to generate simulations of clusters of localizations to model the results from the cross-correlation function. Two separate distributions were generated to simulate dual-channel data, with the parameters for each of the distributions set independently. Seed points for circular domains were chosen at random, with an overall density and number (70 seeds for each channel) determining the number of localizations within each cluster. To replicate the clusters of localizations in each channel, x-y coordinates were selected from a uniform distribution within a predetermined radius from each seed point. A radius of 16 arbitrary units (AU) was chosen to replicate VAMP2, and 12 AU for BoNT/A-Hc, with the number of localizations in the clusters calculated using a density of 0.2/AU^2^. Calculating the cross-correlation while varying the distance between seed points (r = 20, 25, 30 AU) allowed determination of the separation between clusters in each channel that best matched the experimental data. The values obtained from the simulated data (in AU) were converted to nm to enable comparison with the values obtained for experimental data.

## Additional Information

**How to cite this article**: Harper, C. B. *et al*. Botulinum neurotoxin type-A enters a non-recycling pool of synaptic vesicles. *Sci. Rep.* **6**, 19654; doi: 10.1038/srep19654 (2016).

## Supplementary Material

Supplementary Information

Supplementary Movie 1

## Figures and Tables

**Table 1:** Characteristics of protein clusters calculated by fitting auto-correlation values to equation 4.

	**Radius (nm)**	**Increase in molecule density**	**Calculated** #**PSF (nm)**	**Density (per μm**^2^)	**Average molecules per cluster**
Af647-VAMP2	162.1 ± 10.4	1.95 ± 0.07	22.5 ± 1.4	112.2 ± 12.2	18.06
co-labeled Af532-BoNT/A-Hc	120.1 ± 11.2	1.32 ± 0.08	19.6 ± 1.7	220.2 ± 34.1	13.20
Af647-CTB co-labeled	84.9 ± 16.6	2.59 ± 0.33	16.0 ± 1.2	77.3 ± 11.0	4.54
Af532-BoNT/A-Hc	163.3 ± 34.2	0.83 ± 0.09	16.2 ± 1.4	154.4 ± 18.9	10.79

^#^PSF = point spread function, 99% confidence intervals are shown.
