# Supplementary material for: Botulinum neurotoxin type-A enters a non-recycling pool of synaptic vesicles
Source: Sci Rep. 2016 Jan 25;6:19654. doi: 10.1038/srep19654 (PMC4726273; doi:10.1038/srep19654)
Supplement: Supplementary Information [file srep19654-s1.pdf]

Botulinum neurotoxin type-A enters a non-recycling pool of synaptic vesicles

Callista B Harper[1,2], Andreas Papadopulos[1#], Sally Martin[1,3#], Daniel R Matthews[4], Garry P Morgan[5], Tam H Nguyen[1], Tong Wang[1], Deepak Nair[6,7], Daniel Choquet[6] and Frederic A Meunier[1*]

[1]The University of Queensland, Queensland Brain Institute, Clem Jones Centre for Ageing Dementia Research, Brisbane, Queensland 4072, Australia.

[2]Current Address: Centre for Integrative Physiology, University of Edinburgh, Hugh Robson Building, George Square, Edinburgh, Scotland.

[3]Current Address: The University of Queensland, Australian Institute for Bioengineering and Nanotechnology (AIBN), Brisbane, Queensland 4072, Australia

[4]The University of Queensland, Queensland Brain Institute, Brisbane, Queensland 4072, Australia.

[5]The University of Queensland, Centre for Microscopy and Microanalysis, Brisbane, Queensland 4072, Australia.

[6]Interdisciplinary Institute for Neuroscience, The University of Bordeaux, Bordeaux, 33000, France.

[7]Centre for Neuroscience, Indian Institute of Science, Bangalore, 560012, India

*Correspondence to: Frederic A. Meunier, email: f.meunier@uq.edu.au, Tel.: +61-7-3346-6373, Fax: +61-7-3346-6301, Queensland Brain Institute, The University of Queensland, Brisbane, Queensland 4072, Australia.

[#]both authors contributed equally

Supplementary Figure S1: Normalized fluorescence intensity of pHrodo-BoNT/A-Hc in vitro at varying pHs. The data were plotted as mean±sem (*n*=4) and fit to a Henderson-Hasselbach equation ($r^2 = 0.99$), which gave a pKa value of 7.03 (95% confidence intervals = 0.401).

Supplementary Figure S2: NH_4Cl-evoked destaining of presynaptic nerve terminals labeled with pHrodo-BoNT/A-Hc or pHrodo-dextran reduces fluorescence to comparable levels.

Hippocampal neurons (14-17 days *in vitro*) were loaded with either pHrodo-BoNT/A-Hc (300 nM) **(A)** or pHrodo-dextran (0.1 mg/ml) during a 2 min high K^+ stimulation. Neurons were left to recover for 12-15 min prior to the addition of NH_4Cl (50 mM), which alkalizes the lumen of labeled SVs and thus quenches the pHrodo fluorescence. (**A**) Representative nerve terminals labeled with pHrodo-BoNT/A-Hc prior to destaining and 400 s following the addition of NH_4Cl. Enlargements show the response of a representative nerve terminal to the destaining over time. Scale 5 μm. (**B**) Normalized fluorescence of nerve terminals loaded with pHrodo-BoNT/A-Hc (*n*=6) or pHrodo-dextran (*n*=5) in response to NH_4Cl. Data are plotted as mean±sem. (**C**) The change in fluorescent intensity of pHrodo-BoNT/A-Hc was measured *in vitro* in response to pH change from either pH 4 to 8 or from pH 8 to 4 (*n*=3 experiments).

Supplementary Movie S1: BoNT/A-Hc-containing vesicles are distributed throughout hippocampal nerve terminals. A three-dimensional reconstruction of a representative presynaptic region that has endocytosed HRP-BoNT/A-Hc (green vesicles and endosomes). Synaptic vesicles and endosomes that do not contain HRP-BoNT/A-Hc are shown in red. The presynaptic plasma membrane is displayed in yellow. Scale 200 nm.

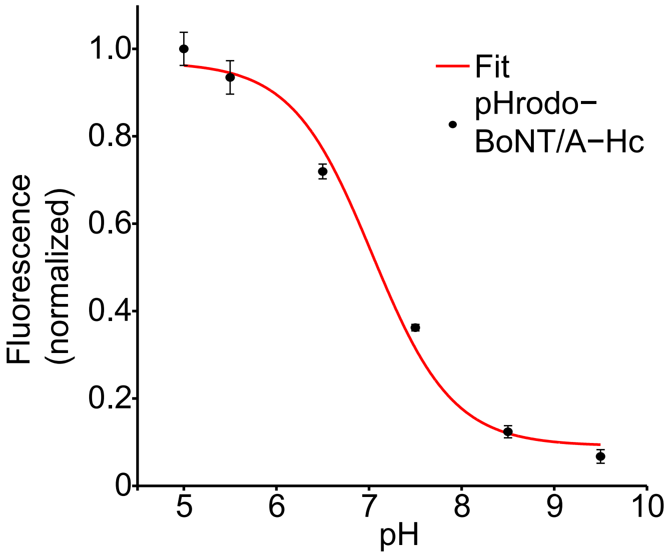

Fit
pHrodo−
BoNT/A−Hc
Fluorescence
(normalized)
1.0
0.8
0.6
0.4
0.2
0
5
6
7
8
9
10
pH

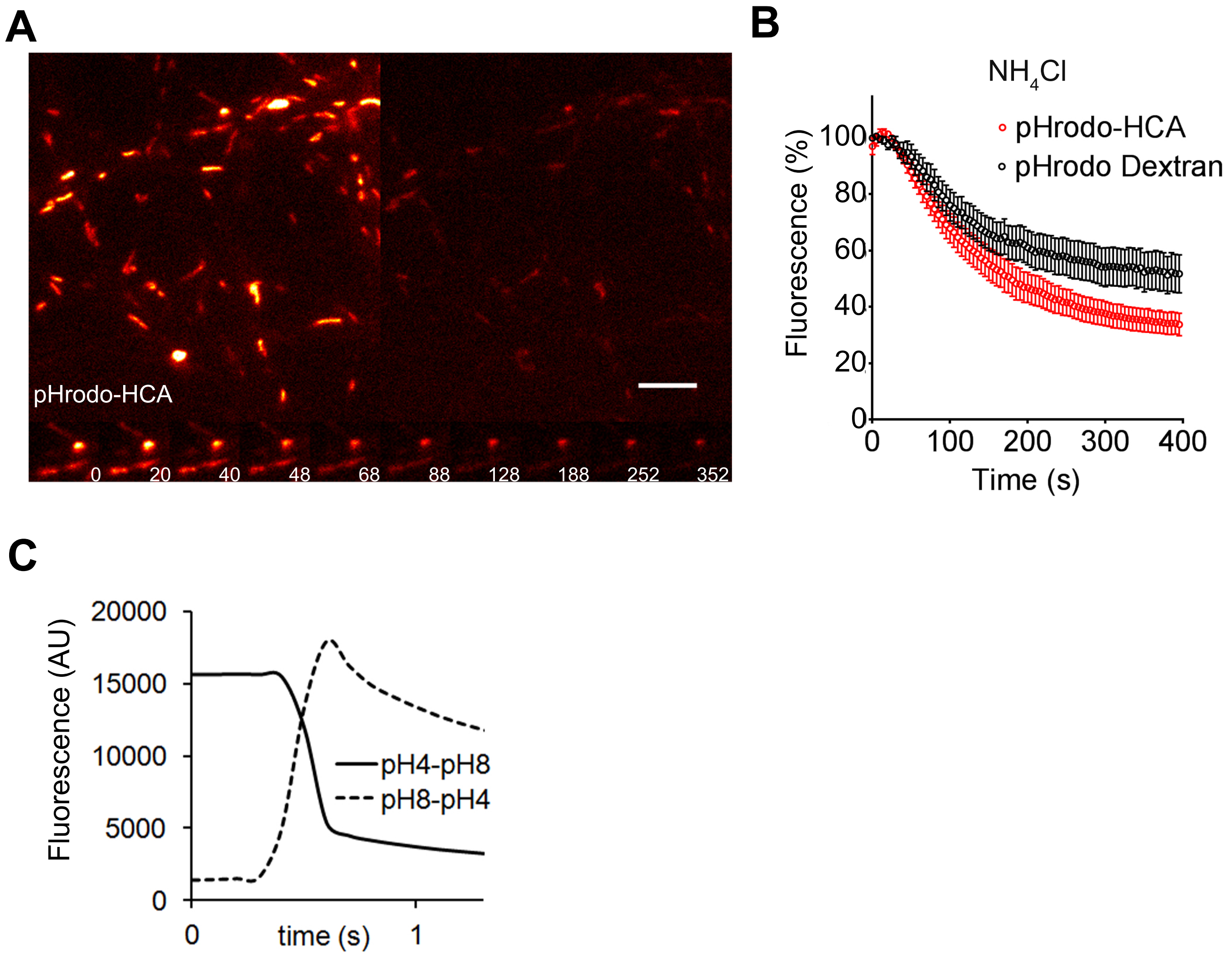
A
pHrodo-HCA
0
20
40
48
68
88
128
188
252
352
B
NH4Cl
pHrodo-HCA
pHrodo Dextran
Fluorescence (%)
Time (s)
C
Fluorescence (AU)
time (s)
pH4-pH8
pH8-pH4
